# Study on the Durability of Graphene Oxide Concrete Composite Under Chloride and Sulfate Environments

**DOI:** 10.3390/ma18194522

**Published:** 2025-09-29

**Authors:** Zhanyuan Gao, Qifeng Shi, Jintao Cui, Jianfeng Lin, Weiting Mao, Marta Kosior-Kazberuk, Julita Krassowska

**Affiliations:** 1 Tianjin Key Laboratory of Structural Protection and Reinforcement for Civil and Construction, Tianjin 300384, China; haifenglingyong@sina.com; 2 College of Civil Engineering, Tianjin Chengjian University, Tianjin 300384, China; sssunnysmile@sina.com (Q.S.); linjianfeng1008@163.com (J.L.); mmwwtt0403@sina.com (W.M.); 3 Department of Building Structures and Structural Mechanics, Faculty of Civil Engineering and Environmental Sciences, Bialystok University of Technology, 15-351 Bialystok, Poland; m.kosior@pb.edu.pl (M.K.-K.); j.krassowska@pb.edu.pl (J.K.)

**Keywords:** graphene oxide concrete composite, Cl^−^ and SO_4_^2−^ solutions, strength reduction rate, mass loss rate, microstructure

## Abstract

In order to study the durability of graphene oxide concrete composite in chloride and sulfate environments, graphene oxide concrete composite specimens were immersed in a mixed solution of 5% sodium sulfate and sodium chloride. After dry–wet cycle immersion and long-term natural immersion, the compressive strength, strength reduction rate, and mass loss rate of concrete specimens were tested. The microstructure was analyzed by scanning electron microscopy (SEM), and the durability of graphene oxide concrete composite in chloride and sulfate environments was analyzed. The results show that with the increase in corrosion age, under dry–wet cycle immersion and long-term natural immersion, the compressive strength reduction coefficient and mass loss rate of graphene oxide concrete composite specimens with 0.07% content are the smallest. The stress–strain curve of concrete after corrosion is flatter than that of uncorroded concrete, and the ductility of concrete specimens after corrosion increased. Through microstructure analysis, it can be seen that the internal structure of graphene oxide concrete composite test block is more compact, the hydration products are regulated, the corrosion of concrete is delayed, and the durability performance is better. Graphene oxide is used to improve the strength and durability of concrete, and the recommended dosage is 0.07%.

## 1. Introduction

Concrete has long been a principal construction material, yet it is highly vulnerable to chloride- and sulfate-laden seawater, groundwater, and soil. Such exposure can rapidly impair both serviceability and safety, precipitating cracking, spalling, or structural collapse and resulting in major human and economic losses [1,2]. Consequently, the durability of concrete in chloride-sulfate environments remains a central research and design priority [3]. Graphene oxide (GO), a graphene-derived nanomaterial, refines cement hydration products and the pore-scale microstructure, thereby markedly enhancing concrete performance [4,5]. Recent studies show that the oxygenated functional groups of GO selectively adsorb Cl^−^, retarding its diffusion into the pore solution [6,7]; this mechanism is expected to synergistically enhance the resistance of GO-modified concrete to coupled chloride-sulfate attack.

To date, the durability of GO-modified concrete exposed to acidic chloride media has been investigated. Zhang et al. [8] used GO dosage as the test variable to elucidate the mechanism by which graphene oxide reduces chloride ingress in recycled-sand ultra-high-performance concrete. Yang et al. [9] incorporated graphene oxide (GO) into cement mortar to quantify its mitigation of ionic erosion. Li et al. [10] incorporated graphene oxide (GO) and polyvinyl alcohol (PVA) into low-cementitious matrices, observing concurrent reductions in porosity, refinement of the pore network, and marked gains in impermeability. Li [11] demonstrated that an optimal dosage of graphene oxide (GO) impedes ingress of Cl^−^ and SO_4_^2−^, thereby minimizing mass loss and mechanical degradation of cementitious composites exposed to saline solutions. Guo et al. [12] examined the durability of GO-modified recycled-aggregate concrete subjected to coupled freeze–thaw cycling and chloride attack, observing that graphene oxide markedly mitigated macroscopic deterioration. Liu et al. [13] explored the effect of graphene oxide (GO) dosage on the resistance of concrete to sulfate attack, demonstrating that even a low dosage significantly enhances sulfate resistance. Muthu et al. [14] quantified the influence of graphene oxide (GO) mass fraction on the resistance of cementitious binders to hydrochloric acid attack. Incorporating GO concurrently reduced mass and cross-sectional-area losses and refined the pore structure. Zhou et al. [15] demonstrated that cement-based composites incorporating medium-grade graphene oxide (GO) exhibit significantly enhanced mechanical strength and corrosion resistance. Devi et al. [16] found that graphene oxide (GO) incorporation densified the concrete microstructure, progressively reducing both carbonation depth and sulfate-induced degradation as GO content increased. Hussain [17] and Yang et al. [18] independently explored sulfate-resisting cement (SRC) blended with sea-sand and seawater as a sustainable marine concrete, demonstrating enhanced chloride binding and microstructural densification. Although graphene oxide (GO) has been shown to mitigate either chloride ingress or sulfate attack individually, its influence on strength loss, mass loss, stress–strain response, and micro-densification in combined chloride-sulfate solutions remains under-studied.

In this study, a mixed Na_2_SO_4_-NaCl acidic solution was used to simulate chloride-sulfate attack on graphene oxide concrete composite (GO-CC) via two exposure regimes: cyclic wetting–drying and prolonged immersion. Subsequent analyses quantified compressive strength, strength-loss rate, mass-loss rate, and microstructural evolution, thereby evaluating the durability of GO-CC in combined chloride-sulfate environments.

## 2. Experiment

### 2.1. Materials and Instruments

Composite Portland cement (CEM I 42.5) was supplied by Tianjin Jidong Cement Co., Ltd. (Tianjin, China). Natural river sand (fineness modulus 2.6; particle size < 2 mm) served as fine aggregate, and crushed granite (5–15 mm) as coarse aggregate. Graphene oxide (GO) was procured from Hunan Fenghua Materials Co., Ltd. (Changsha, China); key properties are listed in Table 1. Analytical-grade anhydrous Na_2_SO_4_ and NaCl (Tianjin Zhiyuan Chemical Reagent Co., Ltd., Tianjin, China) were used as received.

A BILON-500 ultrasonic disperser (10–500 W, Beijing BILON Laboratory Equipment Co., Ltd., Beijing, China), an HJW-60 forced single-shaft mixer (Nanjing New Vitter Testing Instrument Co., Ltd., Nanjing, China), and an RMT-150C servo-hydraulic compression machine (0.01–100 kN s^−1^, Wuhan Institute of Rock and Soil Mechanics, CAS, Wuhan, China) were used for specimen preparation and mechanical testing. Microstructure was examined with a JSM-7800F field-emission SEM (1.2 nm @ 15 kV, 10 nm Au coating, JEOL, Tokyo, Japan).

### 2.2. Preparation of Graphene Oxide Concrete Composite

The GO dosage interval (0–0.09 wt% of cement) was selected for three reasons.

(i) Literature: ≤0.10 wt% GO optimally refines the pore network without significant agglomeration; at higher dosages viscosity increases sharply and air entrainment rises [4,6].
(ii) Pre-screening: pilot compressive-strength tests (5 d, 7 d, and 28 d) peaked at 0.07 wt% GO, corroborating the 27.7% gain reported in Section 3.1.
(iii) Economic factor: at 0.07 wt% GO, the additional binder cost is <2% of the cement price, an increment considered acceptable for marine-grade concrete; hence 0.09 wt% was adopted as the upper bound in this durability study.

To investigate the influence of graphene oxide (GO) on concrete, specimens were prepared with GO dosages of 0%, 0.03%, 0.05%, 0.07%, and 0.09% by cement weight [19,20,21]. GO was preposed at 0–0.09 wt% of cement, dispersed in de-ionized water by ultrasonication (65% rated power, 15 min) and added to the mixer. Homogeneity was ensured by an ice-bath, PCE-assisted pulsed-ultrasonication protocol: GO and 0.05 wt% polycarboxylate superplasticizer (PCE, Mw ≈ 4000 g mol^−1^) were suspended in 50% of the mix water and sonicated for 15 min (20 kHz, 300 W, 40% amplitude, 5 s on/2 s off, 0–4 °C). The suspension was blended with cement within 30 s. Dynamic light scattering (Malvern Zetasizer, Malvern, UK) yielded a GO hydrodynamic diameter of 185 ± 25 nm (PDI = 0.18); UV-Vis analysis (230 nm) showed <5% absorbance loss after 48 h, confirming nano-scale dispersion and stability.

Following GB/T 50080-2016, C30 concrete constituents (Table 2) were batched by mass, introduced to the mixer in the sequence coarse aggregate → fine aggregate → cement, and homogenized. The graphene oxide (GO) suspension and remaining mix water were then added and stirred to produce GO-CC. The fresh composite was cast into 100 × 100 × 100 mm cubes and vibrated until paste flash and bubble cessation. Specimens were demolded after 24 h and standard-cured to 28 d.

Five batches of concrete specimens were fabricated, each with a distinct graphene oxide (GO) dosage: GO0 (0%), GO3 (0.03%), GO5 (0.05%), GO7 (0.07%), and GO9 (0.09%). Every batch contained three replicas, yielding 15 specimens. To examine the influence of immersion regime and corrosion duration, the three immersion protocols were applied at three exposure ages, producing an additional 75 coupons and bringing the total to 90 specimens. All results are reported as the arithmetic mean ± one standard deviation; the coefficient of variation for compressive strength never exceeded 5%.

**Figure 1:**
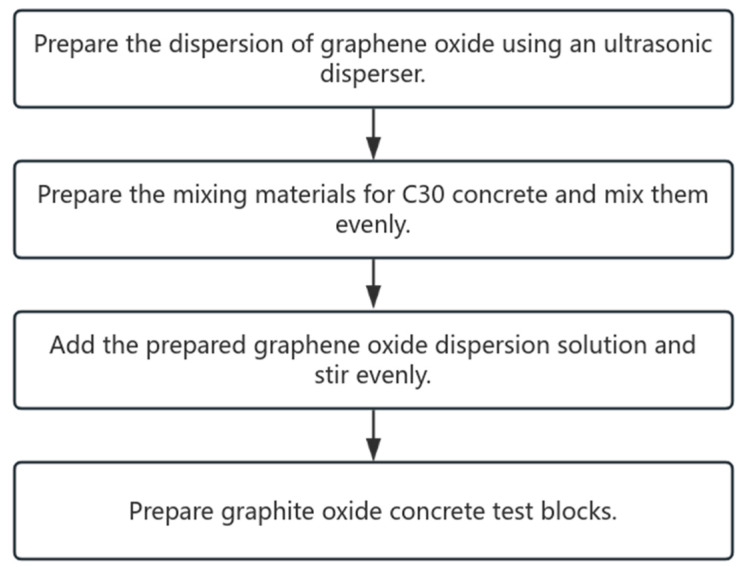
The preparation route map of the graphene oxide concrete composite.

The preparation route map of the graphene oxide concrete composite in this study is shown in Figure 1.

### 2.3. Experimental Scheme

Compressive strength analysis: All tests followed GB/T 50081-2019 (Test Methods for Physical and Mechanical Properties of Concrete): cubic specimens 100 × 100 × 100 mm were loaded at 0.5–0.8 MPa/s. Compressive strength was determined for five graphene oxide (GO) concrete mixes; the reported value for each dosage is the mean of three cubic specimens.

Durability analysis: Graphene oxide concrete cubes were cast and moist-cured at 20 ± 2 °C and ≥95% RH for 28 d in accordance with GB/T 50082-2009. Subsequently, half of the specimens were subjected to cyclic wetting–drying in, and the other half to continuous immersion in, a 5% Na_2_SO_4_ + NaCl solution. The dry–wet cycle immersion lasts three days, followed by three days of natural air drying. Six days is one dry–wet cycle. The solution temperature is controlled at 23 ± 2 °C. The corrosion ages are set at 60 d, 90 d, and 120 d. The long-term corrosion ages for natural immersion are 120 d, 180 d, and 240 d.

After corrosion to the corresponding age, the sample is removed, dried at 105 ± 5 °C until a constant weight is achieved, then weighed, and the mass loss is analyzed. The formula for calculating the mass loss ratio is shown in Equation (1):

(1) $$\Delta m=\frac{m-m_{0}}{m_{0}}\times 100\%$$

In this equation, Δm denotes the mass loss ratio of the graphene oxide concrete composite specimen. $m_{0}$ represents the initial mass of the concrete specimen prior to corrosion, while m indicates the mass of the specimen following corrosion. Both $m_{0}$ and m are determined by averaging the masses of three individual specimens.

Introducing the idea of strength loss rate K, which is described in formula (2) by comparing the average compressive strength $f_{c}$ of GO-CC specimens at various corrosion ages to the average compressive strength $f_{0}$ of regular concrete specimens at the same corrosion age.

(2) $$K=\left|\frac{f_{c}-f_{0}}{f_{0}}\right|\times 100\%$$

Microstructure analysis: A small piece of graphene oxide concrete composite specimen that has corroded to the appropriate age is collected. First, the sample is dried and pasted to the sample holder. Then it is placed in a precision plating apparatus to have gold sprayed on its surface. The gold-plated specimen is placed in a scanning electron microscope for vacuum treatment before being analyzed using SEM technology. Examine the effects of various corrosion ages on the microstructure of concrete.

## 3. Results and Discussion

### 3.1. Compressive Strength Analysis

**Figure 2:**
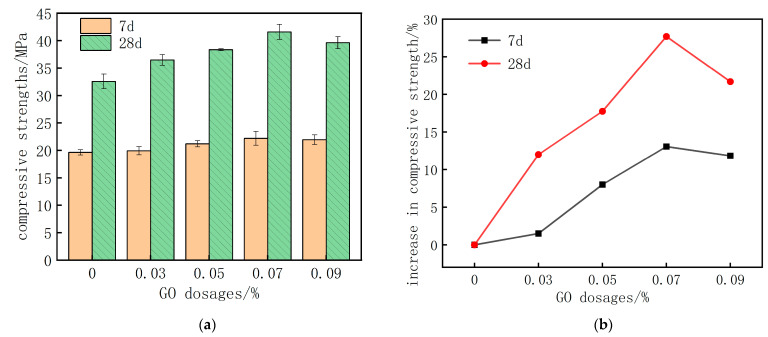
Compressive strength of cement composite with different content of GO. (**a**) compressive strengths value (**b**) increase in compressive strength.

To investigate the impact of chloride and sulfate environments on the compressive strength of GO-CC, first examine the compressive strength of GO-CC that has not been corroded. Figure 2 depicts the compressive strengths and increases of concrete specimens treated with various GO dosages over 7 d and 28 d.

Figure 2 shows that 7 d and 28 d compressive strengths of GO-CC rise with GO content up to 0.07 wt% and decline thereafter. At this optimum dosage, strength increases by 13.1% at 7 d and 27.7% at 28 d, raising the concrete class from C30 to C40 (two strength classes). The GO enhancement is more pronounced at 28 d than at 7 d.

### 3.2. The Impact on GO-CC’s Compressive Strength

#### 3.2.1. GO-CC Compressive Strength

**Figure 3:**
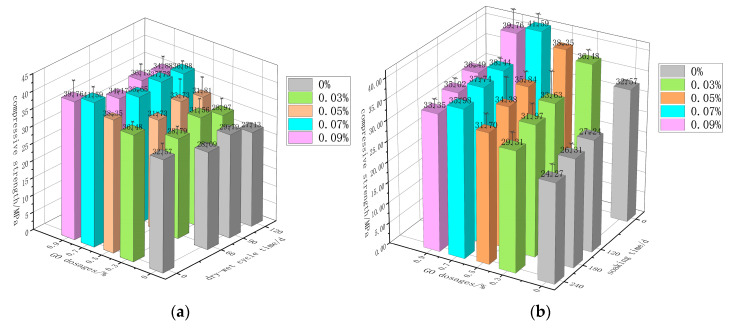
Compressive strength of GO-CC in each bubble cycle. (**a**) dry–wet cycle immersion (**b**) long-term natural immersion.

Following the dry–wet cycle and continuous-immersion regimes detailed in Section 2.3, the post-exposure compressive strength of GO-CC was evaluated; the mean of three cubes per mix is reported in Figure 3. Figure 3 shows that both regimes lowered the compressive strength of GO-CC. Under dry–wet cycling, the loss was virtually uniform across dosages, with the minimum decline recorded at 0.07 wt% GO. Figure 3a shows that, under dry–wet cycling, the compressive strength of GO-CC at every GO dosage first increases and then decreases with immersion time, peaking after 90 d. Figure 3b shows that prolonged natural immersion reduces the compressive strength of GO-CC at every GO dosage.

**Figure 4:**
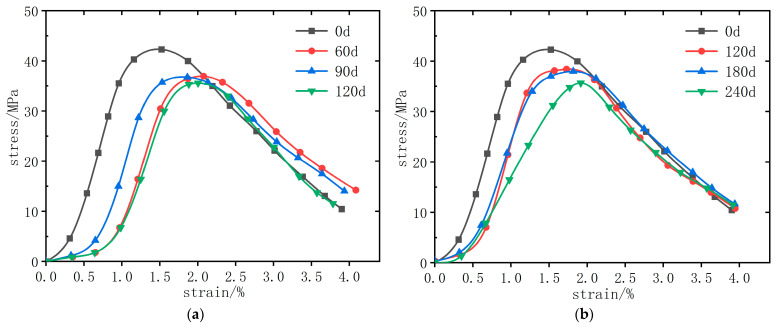
The stress–strain curve of GO content 0.07%. (**a**) dry–wet cycle immersion (**b**) long-term natural immersion.

Stress–strain curves of GO-CC are qualitatively similar across dosages, but the largest enhancement occurs at 0.07 wt% GO; consequently, Figure 4 presents curves for this dosage after both dry–wet cycling and prolonged immersion. The stress–strain curve is separated into two sections: rising and descending. The stress–strain curve variation trends of GO-CC test blocks using the same immersion process are essentially the same. Stress–strain curves for specimens subjected to 60 d or 120 d dry–wet cycles are almost superimposable, with only a marginal extension of the ascending branch. As corrosion age increases, the curve shifts rightward, and the uncorroded specimen exhibits a markedly steeper ascent, evidencing chloride-sulfate-induced micro-cracking. In the descending part, the stress–strain curve of the corroded concrete decreases more slowly than that of the uncorroded concrete, indicating that the corroded concrete specimens have greater ductility.

#### 3.2.2. Strength Loss Rate

**Figure 5:**
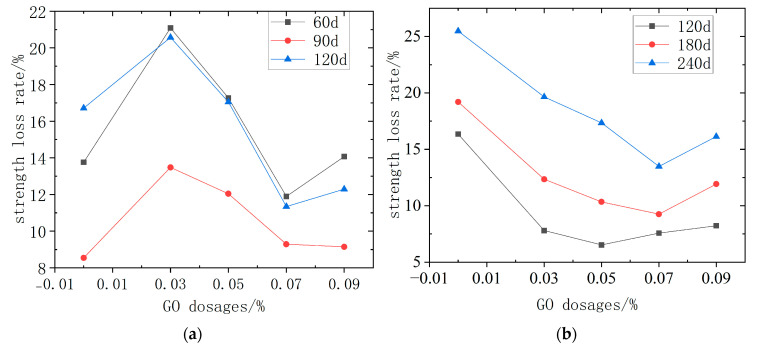
GO-CC strength loss rate. (**a**) dry–wet cycle immersion (**b**) long-term natural immersion.

Figure 5 shows the strength reduction rates of GO-CC under the two immersion settings. Under dry–wet cycling (Figure 5a), the maximum strength loss occurs at 0.03 wt% GO, whereas 0.07 wt% GO exhibits the minimum loss. Moreover, GO-CC specimens soaked for 60 d or 120 d suffer less degradation than plain concrete, indicating that GO has only a marginal influence on strength loss in this environment. Figure 5b shows that in the long-term natural immersion environment, the strength reduction of GO-CC initially drops and then increases. Furthermore, the strength drop rate after adding GO is lower than before adding GO. The lowest concentration occurs when the immersion period is 120 d and the GO dosage is 0.05%. When the immersion duration is 180 d to 240 d, the lowest GO dosage is 0.07%. Adding a certain amount of GO to concrete can improve its internal structure and, to some extent, limit the invasion of hazardous ions. It has been shown that the addition of GO has a considerable effect on concrete strength loss in the long-term natural immersion environment, with the most noticeable benefit occurring at a 0.07% addition quantity.

### 3.3. The Impact on GO Test Blocks’ Mass Loss

#### 3.3.1. Changes in the Appearance of the Specimen

**Figure 6:**
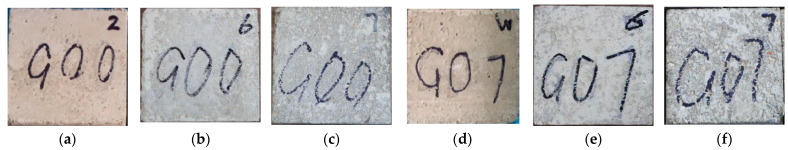
Appearance changes of dry–wet cycle immersion cement composite test block. (**a**) GO0 (60 d) (**b**) GO0 (90 d) (**c**) GO0 (120 d) (**d**) GO7 (60 d) (**e**) GO7 (90 d) (**f**) GO7 (120 d).

After being soaked in the dry–wet cycle and naturally for a long amount of time, the appearance of the GO-CC test blocks altered dramatically. Figure 6 shows the appearance changes of ordinary concrete and GO-CC with a content of 0.07% under dry–wet cycle immersion. Figure 6 shows the appearance changes of ordinary concrete and GO-CC with a content of 0.07% under long-term natural immersion.

Figure 6 illustrates the marked evolution of the surface appearance of concrete specimens subjected to chloride-sulfate dry–wet cycling. At any given age, the GO7 and GO0 specimens exhibit comparable deterioration. As corrosion time increases, the surfaces lose their original smooth finish, become progressively sand-blasted, and suffer edge spalling, culminating in pronounced corrosion marks over the entire block.

**Figure 7:**
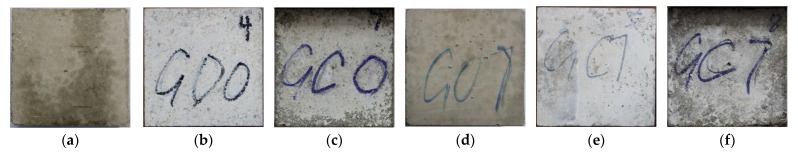
The appearance change of long-term natural immersion cement composite test block. (**a**) GO0 (120 d) (**b**) GO0 (180 d) (**c**) GO0 (240 d) (**d**) GO7 (120 d) (**e**) GO7 (180 d) (**f**) GO7 (240 d).

Figure 7 shows that, during prolonged natural immersion in chloride-sulfate solution, the appearance of concrete specimens remains almost unchanged in the early stage but deteriorates markedly with time. After 180 d, the surfaces of both GO0 and GO7 became uneven and sand-blasted, while at 240 d, peripheral aggregates were exposed and edges exhibited pronounced spalling, indicating severe corrosion.

#### 3.3.2. The Impact of the Quality Loss Rate

**Figure 8:**
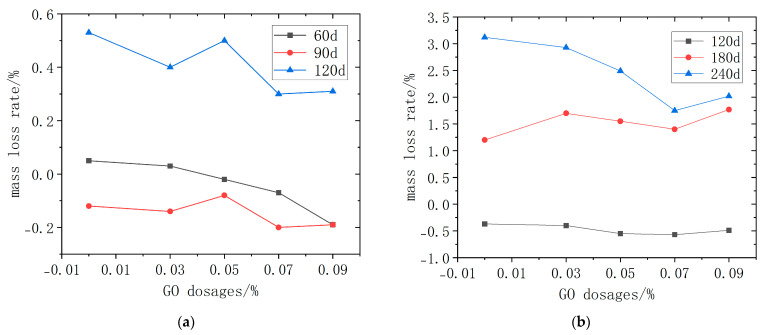
The mass loss rate of GO-CC. (**a**) dry–wet cycle immersion (**b**) long-term natural immersion.

The mass loss of GO-CC test blocks with different dosages after dry–wet cycle immersion and long-term natural immersion is shown in Figure 8. Figure 8a shows that mass change switches from gain to loss as corrosion proceeds; the peak mass occurs at 90 d, where the rate becomes briefly negative. Across all other ages, GO-modified concretes exhibit lower mass-loss rates than the plain control, and—except at 0.05 wt%—the rate decreases monotonically with increasing GO dosage, confirming that GO retards ionic ingress and densifies the microstructure. This beneficial effect is most pronounced under dry–wet cycling. Figure 8b shows that the mass-loss rate accelerates with corrosion age. Between 120 d and 240 d, surface scaling commenced and the rate increased; nevertheless, GO-CC specimens exhibited lower mass-loss rates than plain concrete at all dosages. The minimum rate occurred at 0.07 wt% GO, evidencing superior long-term resistance to combined chloride-sulfate attack.

As shown in Figure 8, the mass rose at the beginning of the immersion. This is attributed to the ingress of sulfate and chloride ions through the pore network, their subsequent participation in cement hydration (forming ettringite and gypsum), and the concomitant pore filling, which increases bulk density. After 90 d of dry–wet cycling, sulfate ions and chloride ions ingress peaked and the maximum mass gain was recorded. As the corrosion age increases, sulfate ions and chloride ions continue to react with the concrete, causing the specimens’ surfaces to peel off and their quality to deteriorate. Meanwhile, the dry–wet cycle causes sulfates and chlorates to crystallize and dissolve in the pores, resulting in the splitting and destruction of internal fissures in the concrete and hastening the chlorate and sulfate erosion response.

### 3.4. Impact on GO-CC’s Microstructure

**Figure 9:**
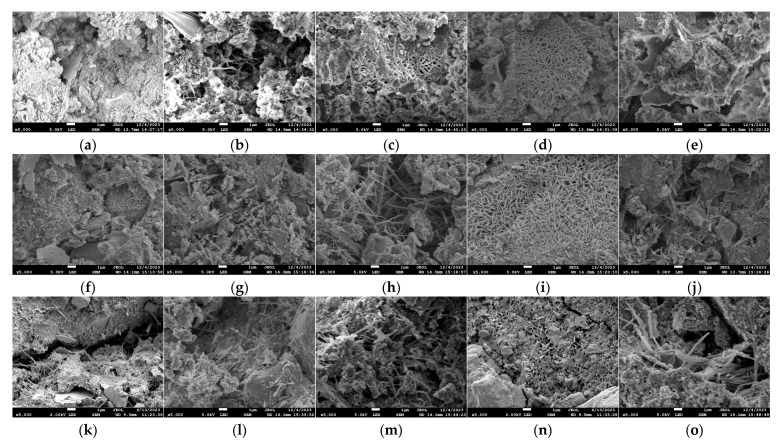
SEM diagrams of the dry–wet cycle bubbled cement composite test block. (**a**) GO0 (60 d), (**b**) GO3 (60 d), (**c**) GO5 (60 d), (**d**) GO7 (60 d), (**e**) GO9 (60 d), (**f**) GO0 (90 d), (**g**) GO3 (90 d), (**h**) GO5 (90 d), (**i**) GO7 (90 d), (**j**) GO9 (90 d), (**k**) GO0 (120 d), (**l**) GO3 (120 d), (**m)** GO5 (120 d), (**n**) GO7 (120 d), (**o**) GO9 (120 d).

The scanning electron microscope (SEM) images of GO-CC specimens with different admixtures soaked in dry and wet cycles for 60 d, 90 d, and 120 d are shown in Figure 9. As can be seen from Figure 9, the microstructure of the blank group following corrosion is not compact. The degree of hydration is low, and there are several holes and cracks inside. Numerous loose and flawed structures are formed by the random distribution of its hydration products, C-S-H gel and C-H. The microstructure of the specimens progressively changed from a significant number of dispersed needle-like and filamentous hydration products to reticular hydration products as the dosage of GO increased. The improvement of the interior structure was most noticeable at a dosage of 0.07%, where the hydration products connected to form a dense microstructure. Later, there was a dense structure locally that was knitted by different hydration products. However, large holes and cracks appeared around it, and a small amount of flocculent C-S-H gel was scattered on the surface.

**Figure 10:**
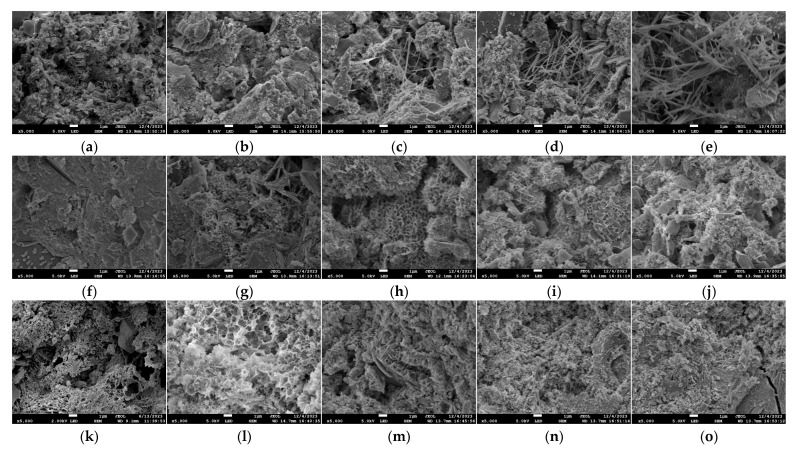
SEM diagrams of GO-CC test block under long-term natural erosion. (**a**) GO0 (120 d), (**b**) GO3 (120 d), (**c**) GO5 (120 d), (**d**) GO7 (120 d), (**e**) GO9 (120 d), (**f**) GO0 (180 d), (**g**) GO3 (180 d), (**h**) GO5 (180 d), (**i**) GO7 (180 d), (**j**) GO9 (180 d), (**k**) GO0 (240 d), (**l**) GO3 (240 d), (**m**) GO5 (240 d), (**n**) GO7 (240 d), (**o**) GO9 (240 d).

The scanning electron microscope images of GO-CC specimens with different dosages after long-term natural immersion are shown in Figure 10. Figure 10 illustrates the hydration products of the microstructure shift from flaky, low crystallinity, and numerous pores to needle-like, networked, and more dense as the GO dosage increases. The hydration products increase dramatically with a dosage of 0.07%. The needle-like and rod-like hydration products penetrate and intertwine, increasing the density of the overall structure. The microstructure of the specimens was loose and not compact, the hydration products reduced, and the holes and gaps increased when the GO level reached 0.09%.

Figure 9 and Figure 10 show that both dry–wet cycling and prolonged immersion allow sulfate and chloride ions to penetrate the pore network, where they react with hydration products to produce expansive phases such as ettringite. These phases initially densify the microstructure by filling accessible porosity; continued formation subsequently generates disruptive tensile stresses once the pore space becomes constrained. Concrete performance therefore declines as soon as this expansion exceeds the critical threshold, and micro-cracks begin to propagate. The concrete specimens exhibit clear corrosion marks after being corroded and immersion. Following corrosion, certain hydration products exhibit ambiguous microstructure morphological traits. The hydration products undergo an uneven distribution and change from a regular microstructure to a loose microstructure. Via its oxygen-bearing functional groups, GO modulates cement hydration, yielding markedly refined reaction products. Pore-scale filling and interfacial cross-linkage densify the matrix and thereby reinforce the concrete. With a high concentration of hydration crystal products, the 0.07% GO specimen has a dense interior structure. It can retain a good microstructure even after immersion in an acidic solution. Therefore, graphene oxide incorporation densifies the concrete microstructure and enhances durability.

**Figure 11:**
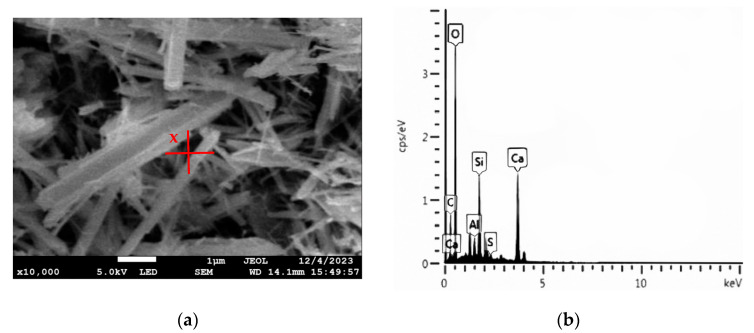
SEM image of GO7 and GO7 x element proportion figure. (**a**) SEM image of GO7 (**b**) GO7 x element proportion figure.

Considering that the combined use of SEM and EDS technologies can simultaneously analyze the morphology and elemental composition of materials at the microscopic scale, in this study, concrete SEM images at a magnification of 5000 times were selected, and combined with EDS technology, quantitative analysis was conducted for the key elements of C, O, Al, Si, S, and Ca. Figure 11 is titled “SEM image of GO7 and GO7 x element proportion figure”.

## 4. Conclusions

Based on the experimental results presented in this study, the following conclusions can be drawn within the scope of this study:

1. Under dry–wet cycling, the compressive strength of GO concrete at every dosage rose to a maximum at 90 d and then declined; under long-term natural immersion, it decreased monotonically. In both regimes, progressive ionic ingress damages the bulk matrix and interfacial zones, leading to systematic strength loss.
2. Under dry–wet cycling, GO confers no measurable reduction in strength loss; by contrast, during long-term natural immersion, it markedly mitigates such loss, with optimum performance at 0.07 wt%. Consequently, GO incorporation is recommended for concrete exposed to long-term chloride-sulfate environments.
3. Regardless of dry–wet cycling or long-term immersion corrosion regime, the mass of GO modified concrete first increases and then decreases with time. GO addition retards acidic-ion ingress; the optimum resistance to long-term attack is achieved at 0.07 wt%, imparting enhanced durability in the latter stages of corrosion.
4. A GO dosage of 0.07 wt% simultaneously maximizes compressive-strength gain and chloride-sulfate durability; this dosage is therefore recommended for concrete enhancement.

The modestly higher optimum dosage (0.07 wt%) relative to the literature values reported under pure sulfate attack (0.05 wt%) indicates that competitive chloride adsorption necessitates a marginal increase in GO. Additionally, the flatter post-peak branches of the stress–strain curves of corroded specimens suggest enhanced ductility attributable to crack branching, warranting further seismic investigation.

The results pertain to unloaded C30 specimens produced with a single commercial GO source; pore-water chemistry, freeze–thaw cycling, and mechanical loading may all influence performance. Future work should therefore encompass long-term field exposure, multi-scale transport modeling, and a life-cycle assessment of GO production to validate and extend these findings.

## Figures and Tables

**Table 1:** Performance parameters of graphene oxide.

Parameter	Number of Layers	Lateral Flake Size & Average Thickness/nm	Purity/%	Moisture/%	Oxygen Content/at.%	Form
Specification	1–3	<5	99	1	45–48	Dry powder

**Table 2:** Mix proportions of graphene oxide concrete composite (kg/m^3^).

Cement	Fine Aggregate	Coarse Aggregate	Water	Water–Cement Ratio
300	672	1248	180	0.6

## Data Availability

The original contributions presented in this study are included in the article. Further inquiries can be directed to the corresponding author.
